# Effects of repeated restraint and blood sampling with needle injection on blood cardiac troponins in rats, dogs, and cynomolgus monkeys

**DOI:** 10.1007/s00580-017-2539-7

**Published:** 2017-08-24

**Authors:** Keisuke Nagata, Kazutoshi Sawada, Hirofumi Minomo, Daisuke Sasaki, Katsuyuki Kazusa, Kazuhiko Takamatsu

**Affiliations:** 1 Drug Safety Research Laboratories, Astellas Pharma Inc., 21 Miyukigaoka, Tsukuba, Ibaraki 305-8585 Japan; 2 Drug Safety Research Laboratories, Shin Nippon Biomedical Laboratories, Ltd., 2438 Miyanoura, Kagoshima, 891-1394 Japan

**Keywords:** Cardiac troponin, cTnT, cTnI, Myocardial injury, Biomarker

## Abstract

While cardiac troponins (cTnT and cTnI) have been used as blood biomarkers of myocardial injury such as myocardial infarction in both humans and animals, their high diagnostic sensitivity inevitably leads to decreased diagnostic specificity. For example, it is difficult to judge whether a slight increase of cardiac troponins in toxicological studies is a treatment-related response or not. Drawing an accurate conclusion requires reliable background data and definitive criteria based on that data. However, no organized efforts in setting such criteria has been reported. Here, we measured blood cTnI and cTnT concentrations in Sprague-Dawley rats, beagle dogs, and cynomolgus monkeys from repeated blood samplings using needle cylinders under restraint up until 24 h after a single oral dose of 0.5 *w*/*v*% methyl cellulose solution as a vehicle. We revealed the extent of individual differences in baseline levels and operational effects. Our results can be useful in making criteria for judgment of treatment-related changes in cardiac troponins.

## Introduction

Cardiac troponins (cTnT and cTnI) are used as clinical blood biomarkers for myocardial injuries such as myocardial infarction (Mahajan and Jarolim 2011) since they have high diagnostic sensitivity and tissue specificity. Since their structure and function are highly conserved across species (O’Brien et al. 2006), cardiac troponins are also used as translational biomarkers in experimental studies in animals (Berridge et al. 2009; Hausner et al. 2013; Herman et al. 2001; Newby et al. 2011; Pierson et al. 2013; Undhad et al. 2012). However, despite the usability of troponins in cardiac injuries, their high diagnostic sensitivity still poses a challenge since increased diagnostic sensitivity inevitably results in decreased diagnostic specificity (i.e., an increased number of false positives) (Mahajan and Jarolim 2011). In particular, when they are applied in toxicological studies, it is often difficult to distinguish treatment-related changes from operational changes. Therefore, obtaining data about blood cardiac troponin levels in intact animals is extremely important.

Schultze et al. previously reported blood cTnI measurements in intact Sprague-Dawley rats and cynomolgus monkeys. Their experiments consisted of careful measurements made over multiple time points under resting conditions after saline administration by oral gavage (Schultze et al. 2009, 2015). Although these studies provided much-needed data for future cTnI research, serial blood samplings were conducted using an automated cannulation method, which is different from the standard procedures of most toxicity studies (i.e., repeated needle injections under restraint).

Here, we aimed to obtain background data in a setting similar to that of typical pharmaceutical toxicological studies conducted in animals. We measured blood cTnI and cTnT concentrations in Sprague-Dawley rats, beagle dogs, and cynomolgus monkeys, from repeated blood samplings using needle cylinders under restraint up until 24 h after a single oral dose of 0.5 *w*/*v*% methyl cellulose solution as vehicle. In addition, for dogs and cynomolgus monkeys, we also measured creatine kinase (CK) and lactate dehydrogenase (LDH) to monitor the extent of struggle during the restraint.

## Material and methods

### Animals experiments

#### Rats

Seven-week-old male and female Sprague-Dawley rats (Crl:CD (SD)) supplied from Charles River Japan Inc. (Tokyo, Japan) were used. Animals were kept in bracket-type stainless steel wire-meshed cages (two or three animals per cage during the study period) at a temperature of 23 ± 3 °C and relative humidity of 55 ± 15% with illumination of 12 h per day from 7 a.m. to 7 p.m. Animals could freely access CRF-1 pellet diet (Oriental Yeast Co., Ltd. (Tokyo, Japan)) and drinking water. Animals were quarantined and acclimated for 2 weeks. Five male and female animals were treated with a single oral dose of 0.5 *w*/*v*% methyl cellulose solution (5 mL/kg) using flexible stomach tubes and syringes. Around 0.25 mL/animal of blood was collected via tail vein while conscious and restrained at 0.5, 1, 2, 4, and 8 h after the treatment. In addition, around 2 mL/animal of blood was collected via the abdominal aorta under anesthesia with isoflurane 24 h after the treatment. Blood samples collected in sodium heparin tubes were immediately placed on ice, centrifuged by 10,000 rpm at 4 °C for 2 min to obtain plasma, and stored at − 80 °C until measurement.

#### Dogs

Ten- to 13-month-old male and female beagle dogs that had been supplied from Hongo Farm, Kitayama Labes Co., Ltd. (Yamaguchi, Japan) were used. Animals were kept in stainless cages (one animal per cage) under the temperature of 23 ± 3 °C and relative humidity of 50 ± 20% with illumination of 12 h per day from 7 a.m. to 7 p.m. Animals were supplied with around 300 g/day of NVE-10 pellet diet (Nihon Pet Food (Tokyo, Japan)) and could freely access to and drinking water. Animals were acclimated to the test condition for 2 weeks, during which the animals were treated with drinking water (30 mL/animal) in the same manner as methyl cellulose solution. After that, 30 male and 30 female animals were treated with a single oral dose of 0.5 *w*/*v*% methyl cellulose solution (5 mL/kg) using disposable catheter and syringe. Around 7.8 (only at − D6) or 2.3 mL/animal per timepoint (0.3 mL for cTnT and 2 mL for the other items) of blood was collected via external jugular vein from conscious animals 6 days before the treatment (− D6) and just before (Pre) and 0.5, 1, 2, 4, 8, and 24 h after the treatment (D0). For the cTnT measurement, blood samples collected in sodium heparin tubes were immediately placed on ice until measurement. For the measurements of the other parameters, collected blood samples were placed at room temperature for 20–60 min, centrifuged (room temperature, 3000 rpm for 10 min) to obtain serum, and either measured within the same day or stored at −70 °C until measurement.

#### Cynomolgus monkeys

Three- to seven-year-old male and female cynomolgus monkeys that had been supplied from Angkor Primates Center Inc. (Kampong Thom, Cambodia) or Tian Hu Cambodia Animal Breeding Research Center Ltd. (Kampong Thom, Cambodia) were used. Animals were kept in stainless cages (one animal per cage) at a temperature of 26 ± 3 °C and relative humidity of 50 ± 20% with illumination of 12 h per day from 7 a.m. to 7 p.m. Animals were supplied with around 108 g/animal/day of HF Primate J 12G pellet diet (Purina Mills, LLC. (MO, USA)) and could freely access to drinking water. Animals were acclimated to the test conditions for 2 weeks, during which the animals were treated with drinking water (10 mL/animal) in the same manner as methyl cellulose solution. After that, 10 male and 10 female animals were treated with a single oral dose of 0.5 *w*/*v*% methyl cellulose solution (5 mL/kg) using disposable catheters and syringes. Around 4.5 (only at − D13) or 2.3 mL/animal per time point (0.3 mL for cTnT and 2 mL for the other items) of blood was collected via femoral vein under unanesthetized condition and restraint in a restraint device 13 days before the treatment (− D13) and just before (Pre) and 0.5, 1, 2, 4, 8, and 24 h after the treatment (D0). For the cTnT measurement, blood samples collected in sodium heparin tubes were immediately placed on ice until measurement. For the parameters of the other items, collected blood samples were placed at room temperature for 20–60 min, centrifuged (room temperature, 3000 rpm for 10 min) to obtain serum, and either measured within the same day or stored at − 80 °C until measurement.

### Dosing formulation

The requisite amount of methyl cellulose (Metlose® SM-400, Shin-Etsu Chemical Co., Ltd., Tokyo, Japan) was dissolved in water for injection (Otsuka Pharmaceutical Factory, Inc., Tokushima, Japan.) to make a concentration of 0.5 *w*/*v*%.

### Clinical testing methods

cTnI and cTnT levels were measured in rats, dogs and cynomolgus monkeys. CK and LDH levels were also measured in dogs and monkeys to monitor the effect by strenuous movement. The measurement methods are as follows.

- cTnI: For rats, plasma samples were measured with Cardiac Injury Panel 3 (rat) Assay Kit and SECTOR® Imager 6000 (Meso Scale Discovery, MD, USA). For dogs and cynomolgus monkeys, serum samples were measured with Multiskan Ascent (Thermo Fischer Scientific, MA, USA).
- cTnT: For rats, plasma samples were measured with Cardiac Injury Panel 3 (rat) Assay Kit and SECTOR® Imager 6000 (Meso Scale Discovery, MD, USA). For dogs and cynomolgus monkeys, blood samples were measured with Cobas h 232 (Roche Diagnostics GmbH, Mannheim, Germany).
- CK and LDH: Serum samples were measured with JCA-BM6070 (Nihon Denshi, Tokyo, Japan) in both dogs and cynomolgus monkeys.

Note that all the testing methods were validated for their intra-assay precision, inter-assay precision, and frozen stability.

## Results

None of the study animals exhibited an abnormal general condition.

### Rats

Plasma cTnI levels were below the lower limit of quantification (BLOQ) at almost all time points except for in one male (RM05) and two females (RF01 and RF02) 2 h after dosing, and one male (RM05) 4 h after dosing (Table 1). The detected levels were from 0.015 to 0.028 ng/mL. All time points for plasma cTnT levels were BLOQ (Table 1).

**Table 1:** Measurements in rats

cTnI measurements in male rats						
	cTnI (ng/mL)					
	0D					
No.	0.5 h	1 h	2 h	4 h	8 h	24 h
RM01	−	−	−	−	−	−
RM02	−	−	−	−	−	−
RM03	−	−	−	−	−	−
RM04	−	−	−	−	−	−
RM05	−	−	0.028	0.015	−	−
Mean	0.000	0.000	0.006	0.003	0.000	0.000
SD	0.000	0.000	0.011	0.006	0.000	0.000
cTnT measurements in male rats						
	cTnT (ng/mL)					
	0D					
No.	0.5 h	1 h	2 h	4 h	8 h	24 h
RM01	−	−	−	−	−	−
RM02	−	−	−	−	−	−
RM03	−	−	−	−	−	−
RM04	−	−	−	−	−	−
RM05	−	−	−	−	−	−
Mean	0.000	0.000	0.000	0.000	0.000	0.000
SD	0.000	0.000	0.000	0.000	0.000	0.000
cTnI measurements in female rats						
	cTnI (ng/mL)					
	0D					
No.	0.5 h	1 h	2 h	4 h	8 h	24 h
RF01	−	−	0.021	−	−	−
RF02	−	−	0.016	−	−	−
RF03	−	−	−	−	−	−
RF04	−	−	−	−	−	−
RF05	−	−	−	−	−	−
Mean	0.000	0.000	0.007	0.000	0.000	0.000
SD	0.000	0.000	0.009	0.000	0.000	0.000
cTnT measurements in female rats						
	cTnT (ng/mL)					
	0D					
No.	0.5 h	1 h	2 h	4 h	8 h	24 h
RF01	−	−	−	−	−	−
RF02	−	−	−	−	−	−
RF03	−	−	−	−	−	−
RF04	−	−	−	−	−	−
RF05	−	−	−	−	−	−
Mean	0.000	0.000	0.000	0.000	0.000	0.000
SD	0.000	0.000	0.000	0.000	0.000	0.000

cTnI: Values below the lower limit of quantification (BLOQ) (0.010 ng/mL) were shown as “–” and regarded as 0 ng/mL in calculation

cTnT: Values below the lower limit of quantification (BLOQ) (0.392 to 0.412 ng/mL) were shown as “–” and regarded as 0 ng/mL in calculation

### Dogs

Serum cTnI levels were detected in almost all animals except for in 2 males (DM22 and DM27) (Table 2). Although the levels detected varied among individuals, a tendency for levels to be constant throughout the examination period was noted in animals that showed higher levels (DM12). For blood cTnT levels, one male (DM26) and five females (DF03, DF13, DF22, DF28, and DF29) showed detectable but lower levels throughout the examination period (Table 2). The other animals showed BLOQ at all points.

**Table 2:** Measurements in dogs

cTnI measurements in male beagles								
	cTnI (ng/mL)							
	− 6D	0D						
No.	−	Pre	0.5 h	1 h	2 h	4 h	8 h	24 h
DM01	−	−	0.19	−	−	0.18	−	−
DM02	−	−	0.19	−	−	−	−	−
DM03	−	−	−	−	0.17	−	0.18	−
DM04	0.16	−	−	0.18	−	−	−	−
DM05	−	−	−	−	−	−	0.40	−
DM06	−	−	0.38	−	−	0.20	0.31	0.25
DM07	−	−	0.19	0.26	0.24	0.16	−	0.26
DM08	0.73	0.71	0.55	0.78	0.41	0.85	1.17	0.58
DM09	0.20	−	−	−	0.17	−	0.25	0.18
DM10	0.35	0.33	−	0.32	0.38	0.43	0.23	−
DM11	−	0.51	0.29	0.53	0.44	0.20	−	0.50
DM12	3.69	3.04	3.16	3.80	3.83	3.79	3.88	3.63
DM13	0.81	0.44	0.44	0.81	0.71	1.04	0.87	0.67
DM14	0.36	0.37	0.31	−	0.58	0.37	0.78	0.42
DM15	−	0.35	−	−	−	0.27	0.25	0.40
DM16	−	−	0.45	0.41	−	−	0.50	0.33
DM17	−	0.32	−	0.38	0.39	−	−	0.32
DM18	0.52	0.37	0.40	−	0.71	0.26	0.36	−
DM19	0.51	0.63	0.57	0.47	0.39	0.90	0.73	0.57
DM20	0.57	0.44	0.57	0.44	0.35	−	0.95	0.38
DM21	−	0.22	−	0.19	−	−	−	−
DM22	−	−	−	−	−	−	−	−
DM23	−	−	−	−	−	0.21	−	−
DM24	−	−	−	−	−	−	0.57	−
DM25	−	0.18	−	−	−	0.29	0.17	0.38
DM26	−	−	−	−	−	−	0.18	−
DM27	−	−	−	−	−	−	−	−
DM28	0.31	0.31	0.20	−	0.35	0.25	0.35	−
DM29	−	0.19	−	−	−	−	−	−
DM30	−	−	0.31	−	−	−	0.39	−
Mean	0.27	0.28	0.27	0.29	0.30	0.31	0.42	0.30
SD	0.68	0.56	0.57	0.70	0.69	0.70	0.72	0.66
cTnT measurements in male beagles								
	cTnT (ng/L)							
	− 6D	0D						
No.	−	Pre	0.5 h	1 h	2 h	4 h	8 h	24 h
DM01	−	−	−	−	−	−	−	−
DM02	−	−	−	−	−	−	−	−
DM03	−	−	−	−	−	−	−	−
DM04	−	−	−	−	−	−	−	−
DM05	−	−	−	−	−	−	−	−
DM06	−	−	−	−	−	−	−	−
DM07	−	−	−	−	−	−	−	−
DM08	−	−	−	−	−	−	−	−
DM09	−	−	−	−	−	−	−	−
DM10	−	−	−	−	−	−	−	−
DM11	−	−	−	−	−	−	−	−
DM12	−	−	−	−	−	−	−	−
DM13	−	−	−	−	−	−	−	−
DM14	−	−	−	−	−	−	−	−
DM15	−	−	−	−	−	−	−	−
DM16	−	−	−	−	−	−	−	−
DM17	−	−	−	−	−	−	−	−
DM18	−	−	−	−	−	−	−	−
DM19	−	−	−	−	−	−	−	−
DM20	−	−	−	−	−	−	−	−
DM21	−	−	−	−	−	−	−	−
DM22	−	−	−	−	−	−	−	−
DM23	−	−	−	−	−	−	−	−
DM24	−	−	−	−	−	−	−	−
DM25	−	−	−	−	−	−	−	−
DM26	−	+	101	+	+	+	+	+
DM27	−	−	−	−	−	−	−	−
DM28	−	−	−	−	−	−	−	−
DM29	−	−	−	−	−	−	−	−
DM30	−	−	−	−	−	−	−	−
CK measurements in male beagles								
	CK (IU/L)							
	− 6D	0D						
No.	−	Pre	0.5 h	1 h	2 h	4 h	8 h	24 h
DM01	134	104	229	97	119	121	122	111
DM02	100	104	161	94	108	139	132	88
DM03	106	107	116	107	133	110	114	101
DM04	102	97	82	74	159	108	186	106
DM05	95	96	152	97	127	128	140	101
DM06	107	96	300	120	86	93	97	96
DM07	105	107	105	97	112	113	115	101
DM08	104	125	198	148	105	109	184	178
DM09	90	91	90	87	92	111	98	91
DM10	97	110	122	127	136	150	146	135
DM11	134	135	184	145	182	163	149	133
DM12	1150	104	90	130	78	106	130	86
DM13	145	102	106	108	129	198	330	113
DM14	108	111	164	108	110	109	105	101
DM15	112	95	100	102	104	103	110	73
DM16	85	451	467	406	352	276	261	75
DM17	103	93	102	125	102	108	150	83
DM18	106	77	94	101	91	97	104	80
DM19	104	110	145	149	164	176	273	114
DM20	83	83	92	107	92	98	112	83
DM21	128	164	178	159	187	219	217	122
DM22	87	203	185	171	208	220	217	94
DM23	99	123	133	132	123	144	344	90
DM24	152	99	103	116	90	107	109	84
DM25	96	102	89	93	96	104	86	80
DM26	80	76	141	80	82	99	102	77
DM27	133	141	152	152	160	171	152	116
DM28	126	127	117	139	136	168	182	100
DM29	83	78	84	87	92	111	127	73
DM30	107	125	125	104	112	134	129	249
Mean	142	121	147	125	129	136	157	104
SD	188	66	77	58	53	44	67	35
LDH measurements in male beagles								
	LDH (IU/L)							
	− 6D	0D						
No.	−	Pre	0.5H	1H	2H	4H	8H	24H
DM01	52	54	47	34	67	45	53	99
DM02	57	43	56	40	63	55	55	50
DM03	52	71	70	64	161	93	89	75
DM04	47	130	66	52	93	76	75	196
DM05	39	42	70	36	92	60	49	46
DM06	61	47	66	50	55	74	51	72
DM07	67	92	90	57	105	98	86	97
DM08	51	44	57	42	53	47	85	94
DM09	58	55	51	91	82	132	72	110
DM10	71	53	62	66	62	93	55	139
DM11	77	61	87	45	137	103	85	102
DM12	127	56	58	120	39	81	85	38
DM13	142	83	44	47	55	134	148	92
DM14	46	43	72	40	37	43	47	55
DM15	88	48	55	47	48	80	79	50
DM16	62	40	68	59	49	40	84	31
DM17	61	54	67	122	44	61	97	53
DM18	45	32	72	90	61	73	55	45
DM19	68	55	82	67	65	67	130	42
DM20	54	42	56	90	52	71	80	47
DM21	79	117	127	74	77	77	60	47
DM22	30	69	74	66	42	50	43	49
DM23	60	86	95	89	45	72	120	29
DM24	100	62	65	93	30	52	34	31
DM25	47	78	38	42	40	49	40	51
DM26	57	63	64	41	46	67	61	42
DM27	56	75	47	51	57	110	50	47
DM28	64	93	39	76	56	60	42	46
DM29	43	41	36	38	38	71	45	42
DM30	52	105	87	38	36	56	42	56
Mean	64	64	66	62	63	73	70	66
SD	24	24	19	24	29	24	27	36
cTnI measurements in female beagles								
	cTnI (ng/mL)							
	− 6D	0D						
No.	−	Pre	0.5 h	1 h	2 h	4 h	8 h	24 h
DF01	0.34	0.75	0.38	0.61	0.75	0.65	0.56	0.50
DF02	−	0.37	0.30	0.33	0.23	0.42	−	0.38
DF03	−	−	−	−	0.29	−	0.33	−
DF04	−	−	−	0.18	−	−	−	−
DF05	−	−	−	−	−	0.22	−	−
DF06	0.51	0.24	0.19	0.23	0.32	0.57	0.38	0.38
DF07	−	−	0.27	−	−	−	0.16	−
DF08	−	−	−	0.17	−	−	0.24	0.26
DF09	−	−	−	0.20	−	−	0.23	−
DF10	0.21	−	0.17	−	−	−	−	−
DF11	−	0.64	0.41	0.35	0.38	0.55	0.29	0.44
DF12	0.38	0.23	0.71	0.50	0.54	0.46	0.52	0.28
DF13	0.22	0.41	−	0.63	0.43	0.56	0.32	0.43
DF14	0.38	0.42	0.62	0.25	0.81	0.54	0.79	0.36
DF15	−	0.44	0.29	0.47	−	0.68	0.46	0.42
DF16	0.27	−	0.46	0.31	0.42	−	0.67	0.34
DF17	−	0.33	−	0.37	−	0.19	−	0.37
DF18	0.55	0.38	0.78	0.26	0.81	0.46	0.65	−
DF19	0.35	0.29	0.22	0.25	−	0.42	−	−
DF20	0.42	0.34	0.31	0.43	0.52	0.23	0.59	−
DF21	−	0.32	−	0.28	−	−	−	−
DF22	−	−	0.42	−	0.29	0.20	0.22	−
DF23	0.32	0.40	0.33	0.65	0.50	0.82	0.39	0.47
DF24	−	−	−	−	0.22	−	−	−
DF25	−	0.16	−	−	−	0.22	0.18	0.21
DF26	−	−	−	−	−	−	0.32	−
DF27	−	−	−	0.21	−	−	−	−
DF28	0.97	0.79	0.88	0.92	0.66	0.84	1.00	0.66
DF29	−	0.23	−	−	−	0.25	−	−
DF30	0.31	0.19	0.38	0.21	0.25	−	0.22	−
Mean	0.17	0.23	0.24	0.26	0.25	0.28	0.28	0.18
SD	0.24	0.23	0.26	0.23	0.27	0.28	0.27	0.21
cTnT measurements in female beagles								
	cTnT (ng/L)							
	− 6D	0D						
No.	−	Pre	0.5 h	1 h	2 h	4 h	8 h	24 h
DF01	−	−	−	−	−	−	−	−
DF02	−	−	−	−	−	−	−	−
DF03	−	−	−	−	−	−	−	+
DF04	−	−	−	−	−	−	−	−
DF05	−	−	−	−	−	−	−	−
DF06	−	−	−	−	−	−	−	−
DF07	−	−	−	−	−	−	−	−
DF08	−	−	−	−	−	−	−	−
DF09	−	−	−	−	−	−	−	−
DF10	−	−	−	−	−	−	−	−
DF11	−	−	−	−	−	−	−	−
DF12	−	−	−	−	−	−	−	−
DF13	−	+	+	+	+	+	−	+
DF14	−	−	−	−	−	−	−	−
DF15	−	−	−	−	−	−	−	−
DF16	−	−	−	−	−	−	−	−
DF17	−	−	−	−	−	−	−	−
DF18	−	−	−	−	−	−	−	−
DF19	−	−	−	−	−	−	−	−
DF20	−	−	−	−	−	−	−	−
DF21	−	−	−	−	−	−	−	−
DF22	−	+	+	+	+	+	+	+
DF23	−	−	−	−	−	−	−	−
DF24	−	−	−	−	−	−	−	−
DF25	−	−	−	−	−	−	−	−
DF26	−	−	−	−	−	−	−	−
DF27	−	−	−	−	−	−	−	−
DF28	−	+	−	−	−	−	−	−
DF29	−	+	+	−	−	−	−	+
DF30	−	−	−	−	−	−	−	−
	CK (IU/L)							
	− 6D	0D						
No.	−	Pre	0.5 h	1 h	2 h	4 h	8 h	24 h
DF01	159	130	226	136	142	157	205	144
DF02	131	129	140	140	136	156	251	125
DF03	104	83	92	86	96	96	97	89
DF04	108	101	93	87	86	96	117	93
DF05	96	78	69	74	89	84	118	83
DF06	96	145	131	102	99	138	118	120
DF07	122	129	105	106	105	111	113	130
DF08	103	148	121	105	93	112	109	153
DF09	111	84	81	78	82	93	112	101
DF10	90	89	90	151	95	110	104	98
DF11	152	160	164	157	158	148	136	155
DF12	119	122	123	106	102	108	106	116
DF13	68	72	66	62	72	63	65	86
DF14	122	112	127	97	113	129	150	115
DF15	112	137	119	115	122	143	346	160
DF16	177	151	243	145	155	153	316	130
DF17	132	141	143	139	129	177	157	113
DF18	141	128	125	125	143	126	144	124
DF19	114	127	96	94	94	105	146	119
DF20	291	153	99	102	108	119	100	88
DF21	83	87	89	87	113	79	79	82
DF22	85	113	80	83	110	98	84	79
DF23	89	79	86	78	88	103	112	80
DF24	122	129	146	121	123	133	132	115
DF25	148	98	115	90	119	117	85	109
DF26	82	83	77	68	86	91	110	76
DF27	86	86	87	91	84	101	95	85
DF28	263	92	127	78	80	91	81	327
DF29	117	165	139	136	135	128	139	103
DF30	103	92	149	213	123	245	108	87
Mean	124	115	118	108	109	120	135	116
SD	48	28	40	32	23	35	64	46
LDH measurements in female beagles								
	LDH (IU/L)							
	− 6D	0D						
No.	−	Pre	0.5 h	1 h	2 h	4 h	8 h	24 h
DF01	66	45	67	42	44	53	80	61
DF02	47	44	43	45	37	59	59	56
DF03	60	55	61	55	56	90	62	65
DF04	49	51	57	46	42	60	82	67
DF05	42	57	36	50	73	71	102	45
DF06	45	47	48	43	41	57	47	54
DF07	60	107	44	38	36	62	56	69
DF08	47	145	38	40	35	59	54	75
DF09	58	53	52	44	46	55	76	55
DF10	52	57	43	57	48	95	78	51
DF11	76	68	65	64	81	56	60	79
DF12	49	43	49	43	43	77	54	59
DF13	42	47	52	37	66	42	46	96
DF14	47	58	84	34	58	78	58	81
DF15	66	108	43	38	57	97	97	88
DF16	94	61	58	49	69	65	100	148
DF17	70	64	50	54	45	150	85	64
DF18	91	81	65	71	129	76	87	82
DF19	93	72	50	47	47	62	121	132
DF20	110	66	48	45	65	93	90	69
DF21	47	53	57	60	154	57	54	69
DF22	41	125	39	43	82	68	37	44
DF23	49	43	72	48	43	63	44	45
DF24	54	87	113	57	50	72	49	71
DF25	54	47	100	43	123	122	34	83
DF26	39	53	42	33	60	52	44	42
DF27	51	51	43	63	38	76	42	45
DF28	75	81	53	46	41	56	33	61
DF29	63	162	66	58	51	54	50	69
DF30	41	40	127	95	52	70	41	46
Mean	59	69	59	50	60	72	64	69
SD	18	31	21	12	28	22	23	24

cTnI: Values below the lower limit of quantification (BLOQ) (0.156 ng/mL) were shown as “−” and regarded as 0 ng/mL in calculation

cTnT: Values below the lower limit of quantification (BLOQ) (50 ng/L) were shown as “−”. Values between 50 and 100 ng/L were shown as “+”

No animals showed abnormal LDH values throughout the examination period (Table 2). One male (DM13 and DM23) and two females (DF15 and DF16) showed higher CK values 8 h after dosing than those at pre-dosing (Table 2). No corresponding change to higher CK values were noted in cTnI or cTnT in these animals.

### Cynomolgus monkey

One female (CF01) showed a higher level of serum cTnI at all points (Table 3). Three males (CM02, CM04, and CM08) and one female (CF09) showed sporadically low levels of cTnI through the examination period (Table 3). Only two males (CM07 and CM08) showed low but detectable blood cTnT values (Table 3). Although the higher levels of CK or LDH were detected sporadically, no correspondences were noted in the changes in cTnI or cTnT levels (Table 3).

**Table 3:** Measurements in cynomolgus monkeys

cTnI measurements in male cynomolgus monkeys								
	cTnI (ng/mL)							
	− 13D	0D						
No.	−	Pre	0.5 h	1 h	2 h	4 h	8 h	24 h
CM01	−	−	−	−	−	−	−	−
CM02	−	−	−	−	−	−	0.20	−
CM03	−	−	−	−	−	−	−	−
CM04	−	0.16	0.26	0.20	−	−	0.17	0.22
CM05	−	−	−	−	−	−	−	−
CM06	−	−	−	−	−	−	−	−
CM07	−	−	−	−	−	−	−	−
CM08	−	−	−	−	−	0.17	−	0.16
CM09	−	−	−	−	−	−	−	−
CM10	−	−	−	−	−	−	−	−
Mean	0.00	0.02	0.03	0.02	0.00	0.02	0.04	0.04
SD	0.00	0.05	0.08	0.06	0.00	0.05	0.07	0.08
cTnT measurements in male cynomolgus monkeys								
	cTnT (ng/L)							
	− 13D	0D						
No.	−	Pre	0.5 h	1 h	2 h	4 h	8 h	24 h
CM01	−	−	−	−	−	−	−	−
CM02	−	−	−	−	−	−	−	−
CM03	−	−	−	−	−	−	−	−
CM04	−	−	−	−	−	−	−	−
CM05	−	−	−	−	−	−	−	−
CM06	−	−	−	−	−	−	−	−
CM07	+	−	+	+	+	+	+	−
CM08	+	−	−	−	+	−	−	−
CM09	−	−	−	−	−	−	−	−
CM10	−	−	−	−	−	−	−	−
CK measurements in male cynomolgus monkeys								
	CK (IU/L)							
	− 13D	0D						
No.	−	Pre	0.5 h	1 h	2 h	4 h	8 h	24 h
CM01	970	328	581	1283	2596	5238	4153	795
CM02	141	263	707	1144	1817	3639	8706	807
CM03	188	216	553	824	951	1823	1779	1004
CM04	399	421	364	512	373	868	959	333
CM05	629	174	1261	715	612	1342	845	310
CM06	140	366	545	1460	268	634	2620	424
CM07	356	302	928	1723	2589	4341	6119	1831
CM08	216	283	385	566	618	1161	2495	870
CM09	458	379	554	1289	1111	1232	2785	577
CM10	179	164	221	524	251	699	520	507
Mean	368	290	610	1004	1119	2098	3098	746
SD	252	82	284	410	860	1586	2458	426
LDH measurements in male cynomolgus monkeys								
	LDH (IU/L)							
	− 13D	0D						
No.	−	Pre	0.5 h	1 h	2 h	4 h	8 h	24 h
CM01	513	457	633	847	707	999	1045	790
CM02	241	447	497	640	740	904	1207	532
CM03	365	495	623	656	899	782	717	579
CM04	618	455	349	733	298	377	341	253
CM05	241	199	298	454	291	441	331	260
CM06	281	355	684	711	407	469	833	327
CM07	298	303	465	581	687	892	990	537
CM08	262	293	403	374	360	513	619	367
CM09	354	343	419	637	508	545	679	372
CM10	236	244	273	544	288	524	345	342
Mean	341	359	464	618	519	645	711	436
SD	122	96	137	130	212	214	296	161
cTnI measurements in female cynomolgus monkeys								
	cTnI (ng/mL)							
	− 13D	0D						
No.	−	Pre	0.5 h	1 h	2 h	4 h	8 h	24 h
CF01	0.72	0.95	0.88	0.77	0.84	0.90	0.83	0.82
CF02	−	−	−	−	−	−	−	−
CF03	−	−	−	−	−	−	−	−
CF04	−	−	−	−	−	−	−	−
CF05	−	−	−	−	−	−	−	−
CF06	−	−	−	−	−	−	−	−
CF07	−	−	−	−	−	−	−	−
CF08	−	−	−	−	−	−	−	−
CF09	−	0.19	−	−	−	−	−	−
CF10	−	−	−	−	−	−	−	−
Mean	0.07	0.11	0.09	0.08	0.08	0.09	0.08	0.08
SD	0.22	0.28	0.26	0.23	0.25	0.27	0.25	0.25
cTnT measurements in female cynomolgus monkeys								
	cTnT (ng/L)							
	− 13D	0D						
No.	−	Pre	0.5 h	1 h	2 h	4 h	8 h	24 h
CF01	−	−	−	−	−	−	−	−
CF02	−	−	−	−	−	−	−	−
CF03	−	−	−	−	−	−	−	−
CF04	−	−	−	−	−	−	−	−
CF05	−	−	−	−	−	−	−	−
CF06	−	−	−	−	−	−	−	−
CF07	−	−	−	−	−	−	−	−
CF08	−	−	−	−	−	−	−	−
CF09	−	−	−	−	−	−	−	−
CF10	−	−	−	−	−	−	−	−
CK measurements in female cynomolgus monkeys								
	CK (IU/L)							
	− 13D	0D						
No.	−	Pre	0.5 h	1 h	2 h	4 h	8 h	24 h
CF01	92	220	160	271	943	2860	1301	286
CF02	125	113	162	691	341	497	837	303
CF03	166	231	225	320	298	545	700	249
CF04	425	1406	2510	2941	4094	7187	4558	1430
CF05	141	213	218	253	293	336	294	257
CF06	317	393	360	569	507	565	964	260
CF07	638	391	1239	1532	1903	1698	2142	550
CF08	290	405	230	439	508	1196	994	381
CF09	255	1512	2180	4080	3935	7108	8755	2369
CF10	190	449	1442	546	572	503	727	316
Mean	264	533	873	1164	1339	2250	2127	640
SD	158	475	859	1250	1412	2555	2494	669
LDH measurements in female cynomolgus monkeys								
	LDH (IU/L)							
	− 13D	0D						
No.	−	Pre	0.5 h	1 h	2 h	4 h	8 h	24 h
CF01	197	239	263	363	391	795	582	265
CF02	386	258	395	444	440	366	345	326
CF03	553	295	387	420	476	421	405	461
CF04	454	636	792	876	962	1462	1053	816
CF05	231	247	258	268	268	299	271	253
CF06	326	288	313	444	352	378	406	306
CF07	331	359	547	627	670	595	564	377
CF08	306	286	285	312	340	448	406	351
CF09	282	468	563	797	772	1011	1038	623
CF10	186	203	298	251	272	289	282	232
Mean	325	328	410	480	494	606	535	401
SD	109	124	165	206	220	360	273	177

cTnI: Values below the lower limit of quantification (BLOQ) (0.156 ng/mL) were shown as “−” and regarded as 0 ng/mL in calculation

cTnT: Values below the lower limit of quantification (BLOQ) (50 ng/L) were shown as “−”. Values between 50 and 100 ng/L were shown as “+”

## Discussion

In this study, we revealed the extent of individual differences in baseline levels and operational effects in Sprague Dawley rats, beagle dogs, and cynomolgus monkeys from repeated blood samplings using needle cylinders under restraint up until 24 h after a single oral dose of 0.5 *w*/*v*% methyl cellulose solution as a vehicle. For the rats, although some animals showed temporal elevations 2–4 h after dosing, cTnI levels were BLOQ at almost all examination points. In contrast, there were substantially larger individual differences in baseline levels of cTnI in dogs (greater than 20-fold) and cynomolgus monkeys (greater than 5-fold). cTnI values fluctuated around individual baselines without clear correlations in timing or with CK and LDH elevations seen in some animals. This suggests that these fluctuations of cTnI values were not caused by the experimental procedures, neither treatment nor operational, and thus individual variations in baseline levels need to be taken into account when evaluating cTnI levels in blood collected. Based on these results, we propose the criteria shown here:

For rats, we can evaluate cTnI levels from blood sampling 24 h after treatment by simply adopting the BLOQ as a criterion for treatment-related effects (e.g., compound-induced effects after drug administration), without needing to consider individual variations or operational effects. When we evaluate cTnI levels from blood sampling collected periodically within the same day of treatment, however, we need to reject temporal elevations as operational effects, based on historical background data defined at each facility (e.g., 0.02 ng/mL, if based on this study).

For dogs and cynomolgus monkeys, we can adopt the following criteria.

1. For all animals in a study, calculate the individual maximum untreated level (IULmax), the individual minimum untreated level (IULmin), and individual untreated range (IULrange; IULmax − IULmin), based on measurements at all-time points for control animals and at time points before treatment for treated animals.
2. Define the criterion of level (CoL) as the highest IULmax and the criterion of variation (CoV) as the highest IULrange in the study.
3. A measured value (MV) taken during the treatment period is considered to have resulted from treatment if MV > CoL and (MV − IULmin) > CoV.

For example, considering Table 3 as data of control animals, CoL and CoV are defined as 0.26 ng/mL (IULmax of CM04 at 0.5 h) and 0.10 ng/mL (the highest IULrange; 0.26–0.16 ng/mL of CM04), respectively. We regarded BLOQ values as 0.16 ng/mL (based on the LOQ value) in this calculation, to avoid overestimating IULrange. Now, suppose that one animal showed a MV of 0.50 ng/mL at some point after treatment and its IULmin was 0.16 ng/mL. In this case, the MV is considered to have resulted from treatment, since MV (0.50 ng/mL) > CoL (0.26 ng/mL) and MV (0.50 ng/mL) − IULmin (0.16 ng/mL) > CoV (0.10 ng/mL).

These criteria could minimize false positives. However, they may not be applied in cases where a small number of animals show considerably higher baseline levels than the others, since inclusion of such animals would lead to underestimation of the treatment-related changes. In such cases, excluding outliers prior to the start of a study could minimize individual variations in baseline levels.

Regarding cTnT, the values were mostly BLOQ, more frequently than those of cTnI. This might be attributable to differences in the measurement systems used. For rats, all cTnT measurements were BLOQ, and therefore, we do not need to consider individual variation or operational effects. For dogs and cynomolgus monkeys, however, we should use the same approach with cTnI, since some animals showed levels exceeding LOQ.

In conclusion, we proposed criteria to distinguish treatment-related effects from individual differences and operational effects in Sprague-Dawley rats, beagle dogs, and cynomolgus monkeys. We admit that our study lacks data from animals after treatment of myocardial infarction-inducing compounds. In the future, such positive control data would be needed and would help us establish more accurate criteria.

## References

[CR1] Berridge BR (2009). A translational approach to detecting drug-induced cardiac injury with cardiac troponins: Consensus and recommendations from the Cardiac Troponins Biomarker Working Group of the Health and Environmental Sciences Institute. Am Heart J.

[CR2] Hausner EA, Hicks KA, Leighton JK, Szarfman A, Thompson AM, Harlow P (2013). Qualification of cardiac troponins for nonclinical use: a regulatory perspective. Regul Toxicol Pharmacol.

[CR3] Herman EH (2001). The use of serum levels of cardiac troponin T to compare the protective activity of dexrazoxane against doxorubicin- and mitoxantrone-induced cardiotoxicity. Cancer Chemother Pharmacol.

[CR4] Mahajan VS, Jarolim P (2011). How to interpret elevated cardiac troponin levels. Circulation.

[CR5] Newby LK (2011). Troponin measurements during drug development—considerations for monitoring and management of potential cardiotoxicity: an educational collaboration among the cardiac safety research consortium, the Duke Clinical Research Institute, and the US Food and Drug Administration. Am Heart J.

[CR6] O’Brien PJ (2006). Cardiac troponin I is a sensitive, specific biomarker of cardiac injury in laboratory animals. Lab Anim.

[CR7] Pierson JB (2013). A public-private consortium advances cardiac safety evaluation: achievements of the HESI Cardiac Safety Technical Committee. J Pharmacol Toxicol Methods.

[CR8] Schultze AE (2009). Longitudinal studies of cardiac troponin-I concentrations in serum from male Sprague Dawley rats: baseline reference ranges and effects of handling and placebo dosing on biological variability. Toxicol Pathol.

[CR9] Schultze AE (2015). Longitudinal studies of cardiac troponin I concentrations in serum from male cynomolgus monkeys: resting values and effects of oral and intravenous dosing on biologic variability. Vet Clin Pathol/Am Soc Vet Clin Pathol.

[CR10] Undhad VV, Fefar DT, Jivani BM, Gupta H, Ghodasara DJ, Joshi BP, Prajapati KS (2012). Cardiac troponin: an emerging cardiac biomarker in animal health. Vet World.

